# Occurrence of tardigrades and morphometric and chemical conditions in rock pools by the Baltic Sea

**DOI:** 10.1038/s41598-023-46697-6

**Published:** 2023-11-13

**Authors:** Sofia Troell, K. Ingemar Jönsson

**Affiliations:** Department of Environmental Science, Kristianstad University, 291 88 Kristianstad, Sweden

**Keywords:** Ecology, Zoology

## Abstract

Rock pools are eroded depressions in bedrock providing temporary aquatic habitats with varying morphometric and chemical conditions. Tardigrades have adapted to many habitats with varying and extreme abiotic conditions, including desiccation, but their occurrence in rock pools have rarely been investigated. This study investigated the occurrence of tardigrades and the morphometric and chemical conditions in rock pools by the Baltic Sea in southeast Sweden. Samples of benthic material were collected from rock pools at three sites near the town Karlshamn together with measurements of pool size, pH, temperature, salinity, and dissolved oxygen of the water. Tardigrades occurred in about one fifth of the rock pools and included five eutardigrade genera. Also rotifers and nematodes were observed in the samples. The morphometric and chemical variables varied both within and among the three sites but with few differences between rock pools with or without tardigrades. However, rock pools with tardigrades tended to be overall shallower than pools without tardigrades, indicating that more desiccating-prone rock pools may be more favourable habitats for tardigrades. The study shows that tardigrades are part of the micro-invertebrate fauna in rock pools and this habitat deserves more investigations into the occurrence of this animal group.

## Introduction

Rock pools are ephemeral aquatic habitats, consisting of depressions in bedrock with occasional water inputs and bottom sediments of trapped unconsolidated material^1^. They occur on rocky outcrops (e.g., granite, limestone, sandstone) in all major biomes from inland areas (freshwater pools) to shorelines (brackish and marine pools) worldwide. Freshwater pools are mainly rainfed^2^, while brackish and marine pools also receive seawater through sprays or waves from a neighbouring sea^3,4^. Rock pools are generally characterized as small-sized habitats with well-defined borders, high local abundance, and as hosts of simple communities and food-webs, making them attractive model systems in ecological and evolutionary research^5–7^. They offer opportunities for both experimental and non-experimental studies about the effects of ephemerality and abiotic stress on biota. Findings in rock pools may also apply to other ephemeral habitats. However, because rock pools often experience some severe abiotic disturbances (e.g., recurring desiccation) and host small populations compared to other habitat types, observed processes and patterns in rock pools must be generalized with care^6^.

The range of hydroregimes^8^ in rock pools is as wide as the global distribution of these habitats, and regional variations may also be considerable. For example, reported hydroperiods (time duration from initial filling to desiccation) averaged from several days to little more than a month in pools located in semi-arid regions of southeast Botswana^9,10^, a few days to about three and a half months in pools of the Korannaberg mountain in central South Africa^11^, and several days to (at least) five months in pools on islands in the archipelago of southwest Finland^12^. Variation, frequency, and periodicity of hydroperiods (that is, hydroregime) sets the boundaries for ecological and evolutionary processes operating in rock pools ^8^. The hydrological selection pressure has resulted in two groups of invertebrate rock pool inhabitants, based on life cycles and dispersal strategies: active and passive dispersers^13^. Most adult active dispersers (e.g., Insecta, Odonata) only thrive in hydrated pools and migrate to other habitats before desiccation occurs^14^, while passive dispersers lack active migratory stages and instead disperse as resting stages via other animals, wind, or overflow of water between pools^1,15^. Passive dispersers represent permanent rock pool inhabitants (so-called ‘rock pool specialists’) with a high extent of endemicity and ability to survive desiccation in situ by adopting drought resistant resting stages^2,14^ such as diapause (suspended development in response to environmental cues), resting eggs or cysts, or anhydrobiotic states (e.g., Crustacea, Nematoda, Rotifera, Tardigrada)^14,16^. This permanent fauna normally possesses additional adaptations to abiotic stresses in rock pools. For example, large diurnal and seasonal fluctuations in environmental conditions are common in the pool environment and impose abiotic stress on biota^2^, especially in shallow pools with low buffering capacity to changes in water variables such as temperature, salinity, pH, and dissolved oxygen^10,17,18^. Shallow pools with short hydroperiods normally support smaller, less competitive species with a high tolerance to stress, whereas deeper pools with long hydroperiods support larger, predatory species with several developmental stages and less tolerance to stress^19–22^, though exceptions to this common observation have been found^10^. Furthermore, freshwater species usually only appear in freshwater pools, while marine species reside in marine pools, and both freshwater and marine species may occur in brackish pools^4,23,24^. However, freshwater-tolerant marine species have also been identified^25^.

Given the variable and ecologically challenging conditions in rock pools, occurrence of the invertebrate phylum Tardigrada in these habitats is of considerable interest. Tardigrades are aquatic micrometazoans inhabiting all major global ecosystems with more than 1400 species described^26^. Many tardigrade species have been adapted to live in highly variable environments with respect to water availability and have evolved an ability to enter a reversible suspension of metabolism called cryptobiosis in response to complete desiccation, defined as anhydrobiosis^27–29^. Other abiotic stress factors suggested to induce cryptobiosis are freezing (cryobiosis), lack of oxygen (anoxybiosis), and high or low salinities (osmobiosis)^27^. Anhydrobiosis results in a high heat tolerance of desiccated tardigrades (up to 100 °C in some species)^30^, while metabolically active (hydrated) tardigrades are considerably more vulnerable to high temperatures (lethal temperature < 40 °C)^31–33^.

Colonisation skill, parthenogenetic reproduction, and resistant resting stages have likely created the basis for the tardigrades’ worldwide dispersal^15^, including extreme environments such as cryoconite holes^34^, deserts^35^, and the abysses of seas^36,37^. They are part of meiofaunal communities in aquatic habitats with all types of water and are also abundant in ephemeral terrestrial and limno-terrestrial habitats (thin water layers on e.g., mosses, lichens, leaf litter, or soils)^14,38^. Occurrence of tardigrades in rock pools has been reported throughout the world (e.g., Israel^39^, Utah, USA^22^, Australia^40^, Ivory Coast^41^, Italy^42^, yet specific studies of tardigrades in rock pools are very scarce. Vecchi et al.^42^ conducted the first study focusing on tardigrades in rock pools and reported a prevalence of tardigrades of 79% in rock pools in an Italian alpine area. The shallowest pools (‘Pans’ < 2 cm depth) had a higher occurrence and taxonomic diversity of tardigrades compared to deeper, less desiccation prone pools (‘Intermediates’ > 2 cm, < 5 cm and ‘Holes’ > 5 cm) in freshwater rock pools of the Italian Apennines. However, tardigrade density was not significantly affected by pool depth and thus hydroperiods. In contrast, Jocqué et al.^22^ observed high densities of tardigrades (*Pseudobiotus* sp.) and rotifers in shallow ephemeral freshwater pools with short hydroperiods on a sandflat in the US state Utah. In other taxonomic groups of invertebrates, such as larger microinvertebrates (e.g., crustaceans^21–23,43^) and macroinvertebrates (e.g., insects^22,23^) densities have been reported to be higher in rock pools with long hydroperiods.

Numerous studies have been reported on animal communities in rock pools related to the Baltic Sea^3,17,44–46^, including the micro- and macrometazoan taxa Rotifera, Nematoda, Turbellaria, Gastropoda, Diptera, Bryozoa, Ostracoda and Crustacea^17,47^. However, there are no previous reports of tardigrades from these shoreline habitats. The purpose of this study was to investigate the occurrence and taxonomic diversity of tardigrades, and the morphometric and chemical conditions in ephemeral rock pools situated by the Baltic Sea in southeast Sweden. Apart from this specific purpose, the study also aims at highlighting a neglected potential habitat for tardigrades found in many different environments around the world.

## Materials and methods

### Site description and selection of rock pools

**Figure 1:**
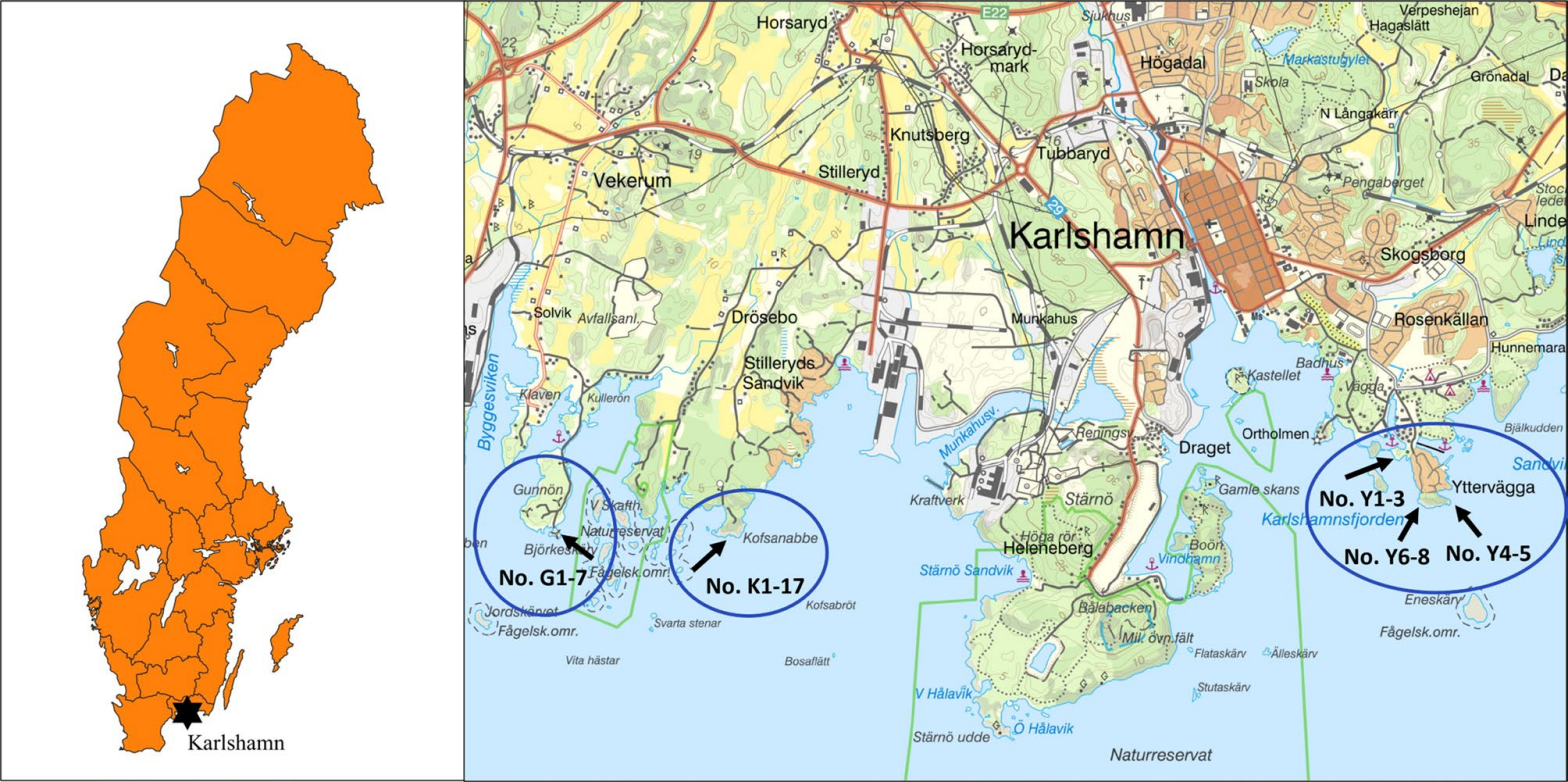
Location of the study area near Karlshamn, Sweden (©2020 SCB), and the three sites of sampling of rock pools at Gunnön (left), Kofsa nabbe (middle) and Yttervägga (right). The location of rockpools within the sites, with reference to pool number, is also shown. Map background: ‘Map 1:50,000’. ©2020 Lantmäteriet/The Swedish Land Survey.

The study was performed in shoreline habitats with exposed rock close to the town Karlshamn at the Baltic Sea in southern Sweden (Fig. 1). The Baltic Sea constitutes an arm of the Atlantic Ocean but with brackish water (ca. 2–8‰). Lack of tide and the low salinity of the adjacent brackish seawater make salinity fluctuations and thus salinity stress on faunal inhabitants rather low in rock pools by the Baltic Sea^17^, although many pools receive inputs of brackish seawater through sprays or by inundating waves^3^. Rain showers, evenly distributed over the year, also dilute the pool waters, often exceeding the evaporation rate that increases salinity. Exclusively rain-filled freshwater pools occur further upshore. The atmospheric temperature directly influences the water temperature of rock pools, creating great annual variations from freezing temperatures during winter to summer temperatures above 30 °C^17^. The predominant wind direction in southern Baltic Sea is west–southwest. Dissolved oxygen content and pH-values are related variables, and closely connected with primary production (mainly algal activity) and community respiration, thus experiencing diurnal and seasonal variations depending on the floral and faunal density and composition of individual pools^44^.

This study investigated rock pools in 1.8–1.7 billon years old granitic and gneissic bedrock along the shorelines of three capes—Yttervägga, Gunnön and Kofsa nabbe—south of Karlshamn (Fig. 1). The geomorphological formations of the rocky shoreline and archipelago of this area, including frequent sites with a multitude of rock pools, date back to the last glacial period (ca. 115–11.5 thousand years ago), and represent ideal conditions for rock pools studies. The rock pools selected at the three sites represented smaller and shallower pools with expected short hydroperiods while large and deep pools were excluded. The selected pools still varied considerably in size (lengths: 30–240 cm; widths: 12–105 cm) and water depths (1.9–29 cm).

### Sampling, extraction and sample analyses

In total 32 rock pools were included in the study. At Yttervägga 8 rock pools were sampled on May 26th in 2021, while at Kofsa nabbe and Gunnön 17 pools and 7 pools, respectively, were sampled on July 9th in 2021. Sampling of benthic material and measurements of the pools were made at the same occasion. All sampling were made at daytime between 9AM and 4PM.

**Figure 2:**
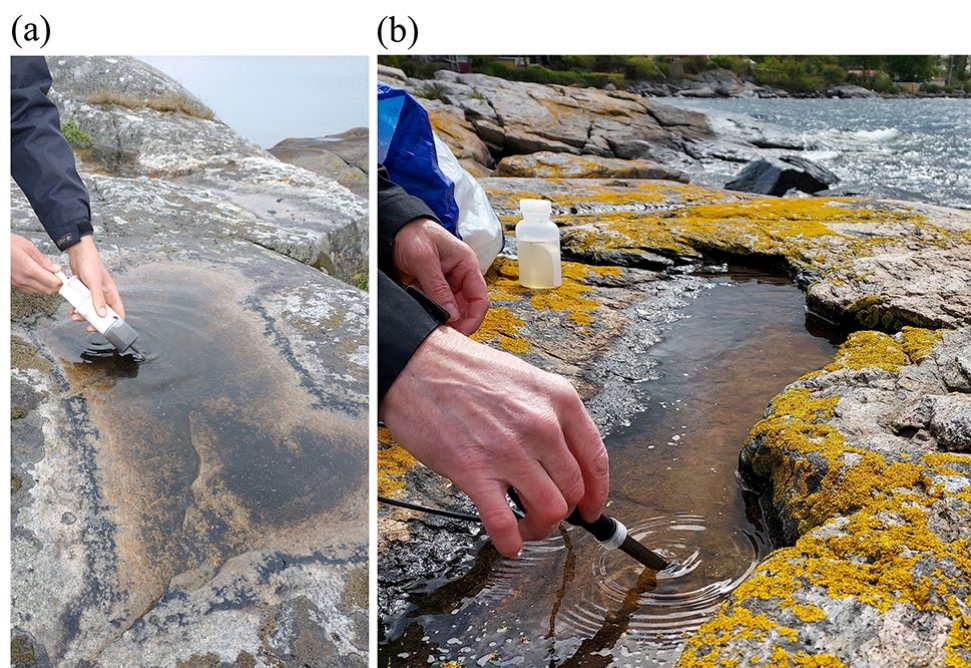
(**a**) Sampling of benthic material from a rock pool at Gunnön. (**b**) Measuring of chemical parameters in a rock pool at Yttervägga. Photos by K. I. Jönsson (**a**) and S. Troell (**b**).

A plastic syringe (100 ml) was used to collect samples of benthic material from different parts of the pools (Fig. 2a), and since benthic material is often concentrated to the deepest parts and bottom crevices of pools these parts were prioritized during sampling. Varying amounts of benthic material were collected from the pools, due to highly different qualities and amounts of benthic material in the pools. Each sample was transferred to a plastic bottle (300 ml) and all bottles were put in a freezer (− 20 °C) in the same day. Analyses of samples took place 1–11 weeks after sampling.

Before extraction of the samples the bottles were thawed in a hot water bath and left standing for a few minutes, allowing the sample material to sediment on the bottom of each bottle. A Pasteur pipette (3 ml) was used to transfer in total 9 ml material from each bottle to petri dishes (3 dishes × 3 ml for bottles containing small or medium amounts of material, or 9 dishes × 1 ml for bottles containing large amounts of or particularly dense material). Distilled water was added to the dishes, which were then analysed for tardigrades under an Olympus SZX9 stereomicroscope, starting at 32 × magnification. The total search time for each dish depended on the absence/occurrence and number of tardigrades found. Every time one or several tardigrades or tardigrade exuvia/eggs were observed, the analysis continued for another 5 mins. If no tardigrades/exuvia/eggs were spotted during the first 5 mins, the analysis was terminated. The upper time limit for one analysis was 90 min. The tardigrades were counted and pipetted to small glass bowls with distilled water and stained with purple-red laceto-aceto-orcein, before mounted on slides in Hoyer’s medium and dried for a few weeks before sealed with transparent nail polish. Mounted specimens were examined under an Olympus BX60 light microscope with up to 1000 × magnification. Tardigrades/exuvia/eggs were photographed with an Olympus UC90 microscope camera and identified to genus level, as it was not possible to identify every specimen to the species level. Taxonomic identification was performed using anatomical descriptions in Nelson et al.^48^, and identification keys in Pilato and Binda^49^ and Bingemar and Hohberg^50^. In addition to tardigrades, the occurrence of rotifers and nematodes in the samples were also documented during the analyses under stereomicroscope, but these animals were not further identified taxonomically.

### Measurements of morphometric and chemical variables

The morphometric rock pool variables (length, width, and maximum water depth) were measured with a tape measure. Pool surface area was calculated using the formula to calculate the area of an ellipse (‘A = a × b × π’, where ‘a’ constitutes the minor radius, and ‘b’ the major radius), because most pools had an oblong, round shape. Chemical water variables (temperature, conductivity, pH, and dissolved oxygen) were also recorded for the sampled pools with a portable HACH-HQ40d multimeter with IntelliCAL LDO101 (temperature and dissolved oxygen), IntelliCAL CDC40 (conductivity), and IntelliCAL PHC201 (pH) probes (Fig. 2b). The equipment failed to record the dissolved oxygen content in one pool at Yttervägga and the conductivity in four pools at Kofsa nabbe, thus data for these pools are missing. All conductivities were converted into salinities with the formula ‘TDS = (EC × 0.65)/1000’ (at temp. 25 °C), where ‘TDS’ is the ‰-amount of ‘Total Dissolved Solids’ in the water and ‘EC’ is the conductivity recorded in µS/cm. The conversion factor 0,65 is based on a TDS/EC-relation in natural groundwaters that are minimally affected by anthropogenic activities^51^.

### Statistical analyses

The potential differences in rock pool size, water depth, and chemical conditions between pools with and without tardigrades (pooling data from all three study sites) were analysed with one-way ANOVA (IBM SPSS Statistics v. 27). No statistical tests were used to compare these two groups within the sites, since the number of rock pools with tardigrades were too small (n < 5) at the sites.

## Results

### Occurrence of tardigrades in rock pools

Tardigrades were found in 19% (6/32) of the sampled rock pools and tended to be more common at Gunnön (57%, 4/7), than at Kofsa nabbe (6%, 1/17), and Yttervägga (13%, 1/8) (Table 1). The highest tardigrade abundance was, however, observed in samples from one of the pools at Yttervägga, with > 100 specimens of the genus *Ramazzottius*, while the other pools with tardigrades had < 10 specimens of various genera. In total, 5 genera from 5 families of the class Eutardigrada were identified. *Isohypsibius* and *Macrobiotus* were the most common genera in the pools with tardigrades (50%, 3/6) and were present in pools at Kofsa nabbe and Gunnön, while *Ramazzottius* was present in pools at Yttervägga and Gunnön. The two remaining genera, *Milnesium* and *Hypsibius*, were only found in pools at Gunnön.

**Table 1:** Tardigrade occurrence in 32 rock pools at three investigated sites (Yttervägga, Kofsa nabbe, Gunnön) by the Baltic Sea.

Site and rock pool no.	Tardigrades/9 ml benthic material	Rotifers	Nematodes
Yttervägga			
Y1		X	
Y2		X	
Y3	*Ramazzottius,* 132 animals, eggs	X	X
Y4		X	
Y5		X	
Y6			
Y7			
Y8			
Kofsa nabbe			
K1		X	
K2		X	
K3			
K4		X	
K5			
K6		X	
K7			
K8	*Isohypsibius,* 4 animals + 3 exuvia with eggs *Macrobiotus,* 2 animals 1 animal undetermined	X	
K9			
K10			
K11			
K12		X	
K13		X	
K14			
K15			
K16			
K17			
Gunnön			
G1	*Isohypsibius,* 1 animal	X	
G2	*Isohypsibius,* 2 animals, 1 exuvium *Hypsibius,* 2 animals *Macrobiotus,* 1 animal 2 undetermined animals	X	X
G3	*Macrobiotus,* 1 animal	X	
G4		X	
G5	*Milnesium,* 1 animal *Ramazzottius,* 6 animals	X	
G6			
G7		X	X

Tardigrade genera, total number of animals and exuvia, presence of eggs, and presence of rotifers and nematodes in the pools are presented. “Undetermined” animals represent specimens lost during slide preparation. Proportions of rock pools with tardigrades at each site (with 95% confidence limits): Yttervägga, 0.13 (0.022–0.47); Kofsa nabbe, 0.059 (0.011–0.27); Gunnön, 0.57 (0.25–0.84).

Rotifers occurred in several pools at all three sites (56%, 18/32) and were often abundant in the samples, while nematodes were present in few pools (9%, 3/32) at Yttervägga and Gunnön and generally rare in the samples. All six pools with tardigrades also contained rotifers and two of them contained nematodes, while one pool contained both rotifers and nematodes but no tardigrades.

### Relationship between tardigrade occurrence and rock pool conditions

The morphometric and chemical conditions of all rock pools are presented in Supplementary Table S1, and statistical summaries for pools with and without tardigrades at the three sampled areas are given in Table 2. Combining the data from all sites, rock pools with tardigrades were overall significantly shallower (4.6 ± 2.8 cm) compared to the pools without tardigrades (11.1 ± 7.3 cm) (F_1,30_ = 4.47, P = 0.043), and tended to have smaller surface area (0.2 ± 0.2 m^2^) than pools without tardigrades (0.5 ± 0.4 m^2^) but not significantly so (F_1,30_ = 1.78, P = 0.19). The recorded estimates of temperatures and dissolved oxygen were similar in pools with and without tardigrades at all three sites, and combining data from all sites there were no significant differences between pools with tardigrades and pools without tardigrades (temperature: F_1,30_ = 0.011, P = 0.92; oxygen: F_1,29_ = 3.74, P = 0.063).

**Table 2:** Surface areas, water depths, and chemical conditions of 32 rock pools at the three investigated sites (Yttervägga, Kofsa nabbe, Gunnön) by the Baltic Sea.

Yttervägga	With tardigrades (N = 1)				Without tardigrades (N = 7, only 6 for oxygen)			
					Avg	SD	Min	Max
Surface area (m^2^)	0.01				0.18	0.19	0.05	0.56
Water depth (cm)	2.0				15.9	8.0	8.5	29.0
Temperature (°C)	16.2				15.8	0.7	14.6	16.6
Salinity (‰)	0.01				3.69	3.16	0.06	8.58
pH	7.9				8.3	0.8	7.1	9.6
Oxygen (mg/l)	10.10				9.34	1.76	7.42	12.64

Kofsa nabbe	With tardigrades (N = 1)				Without tardigrades (N = 16, only 12 for salinity)			
					Avg	SD	Min	Max
Surface area (m^2^)	0.10				0.53	0.44	0.13	1.79
Water depth (cm)	3.2				9.5	6.9	1.9	21.8
Temperature (°C)	22.9				22.4	1.5	20.2	25.7
Salinity (‰)	0.39				1.85	3.59	0.07	11.97
pH	8.9				8.8	1.0	6.7	9.6
Oxygen (mg/l)	11.43				12.95	2.20	9.78	17.42

Gunnön	With tardigrades (N = 4)				Without tardigrades (N = 3)			
	Avg	SD	Min	Max	Avg	SD	Min	Max
Surface area (m^2^)	0.31	0.20	0.19	0.61	0.64	0.21	0.43	0.85
Water depth (cm)	5.6	3.0	2.7	9.8	8.7	4.6	3.6	12.5
Temperature (°C)	20.9	0.58	20.1	21.5	21.6	0.8	21.0	22.5
Salinity (‰)	0.04	0.01	0.03	0.04	0.04	0.01	0.04	0.05
pH	6.7	0.5	6.0	7.2	8.1	0.9	7.1	8.7
Oxygen (mg/l)	9.20	0.45	8.61	9.70	10.36	0.60	9.70	10.87

Statistics for pools with and without tardigrades are shown. Averages (Avg.), standard deviations (SD), minimum and maximum values (min. and max.) are presented. Estimates of dissolved oxygen of one pool at Yttervägga, and salinities of four pools at Kofsa nabbe are missing, due to problem with the portable multi meter during measuring (see Supplementary Table S1).

All pools with tardigrades were freshwater pools (salinity < 0.5‰). The pools without tardigrades, on the other hand, showed greater variation in their salinities and contained both freshwater and brackish water (0.04 ± 0.006‰ at Gunnön, 1.8 ± 3.6‰ at Kofsa nabbe, and 3.7 ± 3.2‰ at Yttervägga). However, there was no significant difference in salinity between pools with and without tardigrades (F_1,26_ = 2.31, P = 0.14).

A large range of pH-values below and above neutral pH 7.0 were found in the pools at the respective sites. The pH-values of the single pools with tardigrades at Kofsa nabbe (pH 8.9) and Yttervägga (pH 7.9) were close to the average pH-values of the pools without tardigrades (8.8 ± 1.0 at Kofsa nabbe, 8.3 ± 0.8 at Yttervägga), while the average pH-values differed 1.4 units between pools with (6.7 ± 0.5) and without (8.1 ± 0.9) tardigrades at Gunnön. Overall, pools with tardigrades had significantly lower pH than pools where no tardigrades were found (F_1,30_ = 8.36, P = 0.007).

## Discussion

### Occurrence of tardigrades

This study is the second that specifically investigated the occurrence of tardigrades in rock pools (following the first by Vecchi et al.^42^), and the first to document tardigrades in rock pools by the Baltic Sea. Tardigrades were found in pools at all three sites and in nearly 20% of the samples, suggesting that tardigrades represent a relatively common component of the meiofaunal communities and biodiversity of rock pools of this archipelagic region. The prevalence of tardigrades in this study is however considerably lower than in the study by Vecchi et al.^42^ of Italian alpine rock pools (79%). In all except one of the pools in our study the number of specimens found was low, but the benthic material analysed from each pool only represented a small proportion of the total material in the pools, making it likely that the total number of tardigrades in the pools were higher. The presence of exuvia and eggs also indicates that these tardigrades have resident populations in the rock pools.

Rotifers were more common and abundant in the pools, while nematodes were few and less common than tardigrades. Both rotifers and nematodes have been reported from Baltic rock pools before, but nematodes were found much less frequently in these pools^17,47^, in line with the results of this study. Nematodes in rock pools on islands of the southern archipelago of Finland appeared to be immigrants to the pools from nearby intertidal algae, terrestrial moss, or grass carpets^47^.

In the present study the pools hosted a diversity of eutardigrade genera, indicating tardigrade dispersal to the pools from nearby limnic or limno-terrestrial habitats (*e.g.*, lichens, soils, grass turfs). The idea of short-distance dispersal from surrounding habitats to rock pools is supported by Jensen^47^ for nematodes, and also by Vecchi et al.^42^ who found a close correspondence between the examined tardigrade community composition of freshwater rock pools in the Italian Apennines and the community composition previously recorded in the same region for mosses, lichens, and freshwater habitats.

Many tardigrades, rotifers and nematodes possess resistant resting stages and an ability to colonise rapidly (e.g., by wind dispersal) and reproduce asexually by parthenogenesis^15^, which are common characteristics of passive dispersers^2,13,14^ and especially favoured in ephemeral habitats such as rock pools. Drought resistant resting stages^16,27–29^ allow these phyla to withstand recurring desiccation, which excludes more drought-sensitive taxa from ephemeral pools (e.g., species of Insecta or Odonata^14^). Our finding of a higher occurrence of tardigrades in shallow Baltic rock pools compared to deeper pools is consistent with earlier records of tardigrades in freshwater pools^22,42^. This pattern may relate to the ability of tardigrades to survive severe and recurrent desiccation by anhydrobiosis. Larger, predatory invertebrates generally inhabit deeper pools with longer hydroperiods that allow longer life cycles of species^19–23,43^. These potential predators and competitors of tardigrades are thus excluded from shallow rock pools with short hydroperiods and exposure to dry conditions, which may create more favourable conditions for tardigrades to persist and proliferate.

### Morphometric and chemical conditions

Large diurnal and seasonal fluctuations in morphometric and chemical conditions are often recorded in ephemeral rock pools with a low buffering capacity to changes in the pool environment^2,10,17,18^. The measurements performed in this study only provide a snapshot of the conditions of the investigated pools, since the study was restricted to just one day of field measurements at each of the three selected sites. To get a more detailed picture of how pool conditions fluctuate over time, monitoring of pools during several hydroperiods is necessary. However, recordings of many pools at a single time can still provide information on natural variation in pool conditions among different rock pools. In our study, the existence of such variation was clear for salinity, pH, and dissolved oxygen.

Baltic rock pools are considered to have low salinity fluctuations due to the low salinity of the adjacent brackish seawater that may reach pools through sprays or by inundating waves^3,17^. Recurring rainfalls over the year also counteract evaporation and dilute the water in pool by the Baltic Sea. This study mainly recorded low salinities that represented freshwater in the pools, yet some of the pools at Yttervägga and Kofsa nabbe contained brackish water. Tardigrades only occurred in freshwater pools at all three sites, and this may be expected since genera of mainly limnic and limno-terrestrial tardigrades were identified, and freshwater invertebrates usually only appear in freshwater pools^23,24^. Some limno-terrestrial tardigrades have been documented for a high tolerance to osmotic stress and may well survive saline conditions^52^, but whether they may sustain populations under low saline/brackish conditions is unknown, and the presence of tardigrades in the brackish water of the Baltic Sea is largely unexplored.

The temperate climate of the Baltic region makes annual variations in the atmospheric temperature large and constitute one of the main regulating factors of Baltic rock pool communities^17^. The recorded pool temperatures in this study were close to the ambient air temperature and similar in the pools at the respective sites, around 16 °C at Yttervägga in May, and 20–23 °C at Kofsa nabbe and Gunnön in July. Despite a tolerance of tardigrades to temperatures up to 100 °C in the desiccated anhydrobiotic state^30^, under active hydrated conditions tardigrades do not tolerate temperatures over 40 °C^31–33^. Summer temperatures of Baltic Sea rock pools may exceed 30 °C^17^ and may well reach hazardous levels for tardigrades under heat waves, especially in small and shallow rock pools. Low temperatures, on the other hand, is likely to be much less of a problem for tardigrades inhabiting rock pools since many tardigrades are able to withstand very low freezing temperatures^53^. Shallow rock pools are more likely to freeze to the bottom during the winter than deeper ones, and tolerance to freezing is therefore a requirement of invertebrates that maintain permanent populations in Baltic Sea rock pools, particularly in the northern parts of the sea. Interestingly, in a recent study on tolerance to repeated freeze–thaw cycles in 12 different tardigrade species from different habitats, specimens of the genus *Ramazzottius* collected from Italian mountain rock pools were reported to survive the highest number of cycles^54^, indicating that tardigrades living in habitats with frequent freeze–thaw and/or desiccation-hydration cycles such as rock pools are also well adapted to survive such conditions.

Small ephemeral rock pools are also characterized by large diurnal and seasonal variations in dissolved oxygen content and pH, which mainly depend on primary production and community respiration in pools^10,17,44^. Although levels of dissolved oxygen varied considerably among rock pools in this study, pools with and without tardigrades did not differ significantly in oxygen level, while pH values were significantly lower and closer to neutral pH 7.0 in rock pools with tardigrades compared to pools without tardigrades. The limited scope of our study with relatively small number of rock pools investigated (limiting the power of the statistical tests) does not allow us to draw any firm conclusions from these results, but both oxygen levels and pH have been reported to show considerable variation at a daily basis in Baltic Sea rock pools^17^, which makes it worthwhile to more closely investigate how tardigrades respond to variation in these factors.

## Conclusion

In conclusion, this study shows that tardigrades are part of the meiofauna in rock pools of the Baltic Sea shoreline, although present in only one fifth of the rock pools. Rock pools where tardigrades were found tended to be shallower and have lower pH, while other measured parameters did not show a relationship with presence of tardigrades. Invertebrate communities of rock pools deserve more studies in view of the extreme variability of abiotic conditions in this extreme habitat, and tardigrades represent a particularly interesting organism to investigate due to its documented tolerance to some of the extreme conditions that rock pools provide. Rock pools offer a range of opportunities for both descriptive and experimental studies on responses to abiotic stress, population dynamics, and dispersal of tardigrades in this small-scale natural habitat type that occurs in many different ecosystems. Due to their small scale and well-defined borders, rock pools are easy to measure and manipulate, and their abundance and morphometric and chemical variability make them ideal as model systems for ecological and evolutionary research.

### Supplementary Information

Supplementary Table S1.

## Data Availability

All data underlying this article are presented in the article and in its online supplementary information.
